# Supplementary material for: Heterologous mogrosides biosynthesis in cucumber and tomato by genetic manipulation
Source: Commun Biol. 2023 Feb 17;6:191. doi: 10.1038/s42003-023-04553-3 (PMC9938114; doi:10.1038/s42003-023-04553-3)
Supplement: Supplementary file 2 — Supplementary Information [file 42003_2023_4553_MOESM2_ESM.pdf]

Supplementary information

Supplementary Fig. 1 Schematic diagram of mogrosideV (in the blue box) and cucurbitacin C (in the red box) biosynthesis in the fruits of *S. grosvenorii* and *C. sativus*. Mogrosides biosynthesis genes were cloned and assembled into pCAMBIA1300 vector are shown in blue. *SgSQE1*, squalene epoxidase; *SgCS* and *CsBi*, oxidosqualene cyclase; *SgEPH2*, epoxide hydrolase; *SgP450* and CYP450, cytochrome P450 mono-oxygenase; *SgUGT269-1* and *SgUGT289-3*, UDP-glucosyltransferases; ACT, acyltransferase.

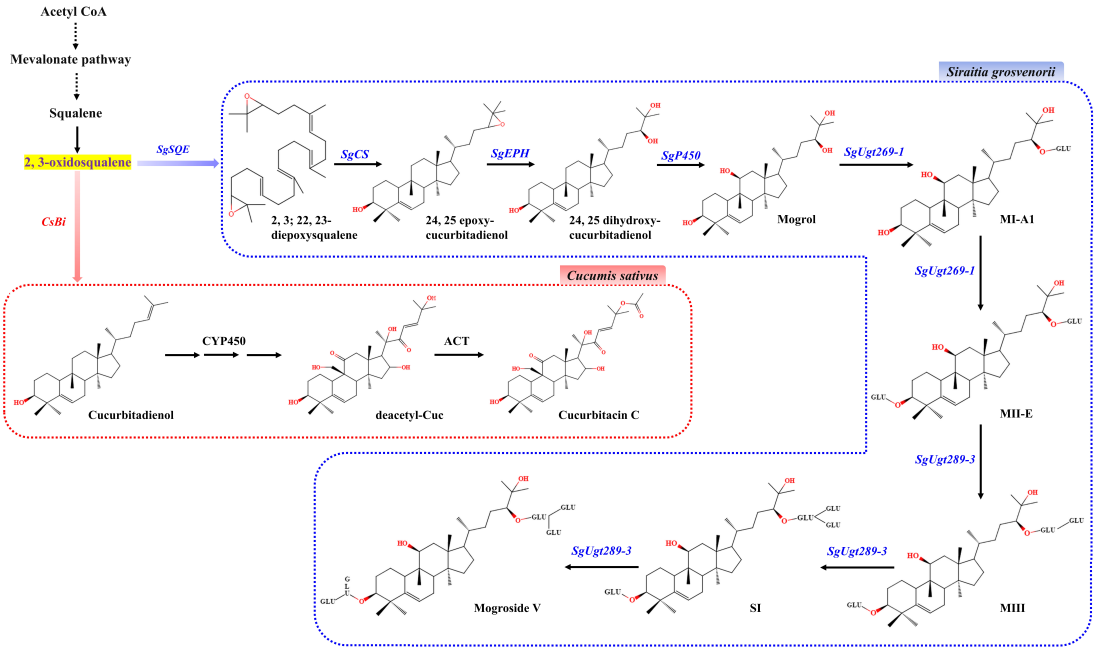

Supplementary Fig S2. Construction of multigene vector with 6 mogrosides biosynthesis genes in pCAMBIA1300. a) Flowchart of multigene cassette assembly. The amino acid sequence of P2A is GSGATNFSLLKQAGDVEENPGP. *Bam*HI, *Sac*I, *Hin*dIII and *Eco*RI are restriction enzyme sites. **b)** Amplification of specific fragments of the *SgSQE1* (1587 bp), *SgCS* (2280 bp), *SgEPH2* (951 bp), *SgP450* (1421 bp), *SgUGT269-1* (1253 bp), *SgUGT289-3* (1026 bp) and Hyg (392 bp) genes from the U22p-SCE vector. Included is a DNA marker (4.5 kb), and the red lines indicate specific sizes of marker bands.

a

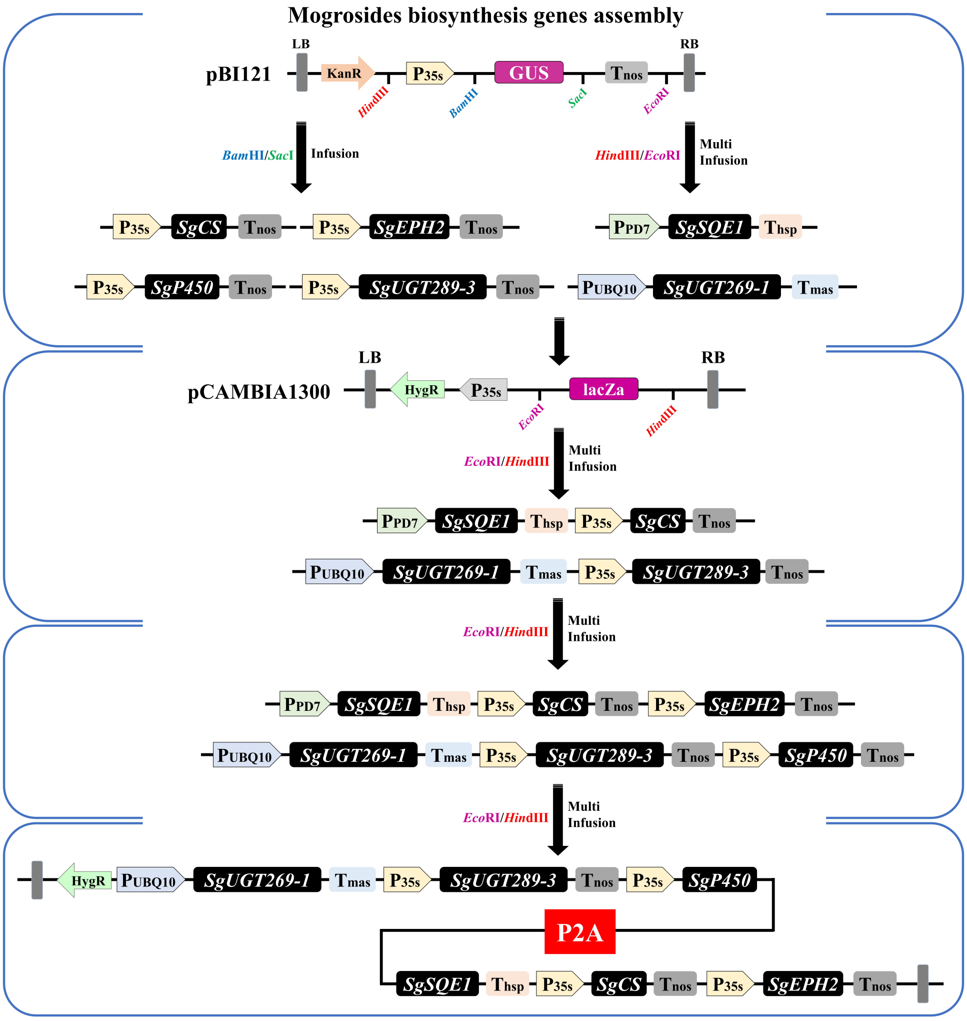

b

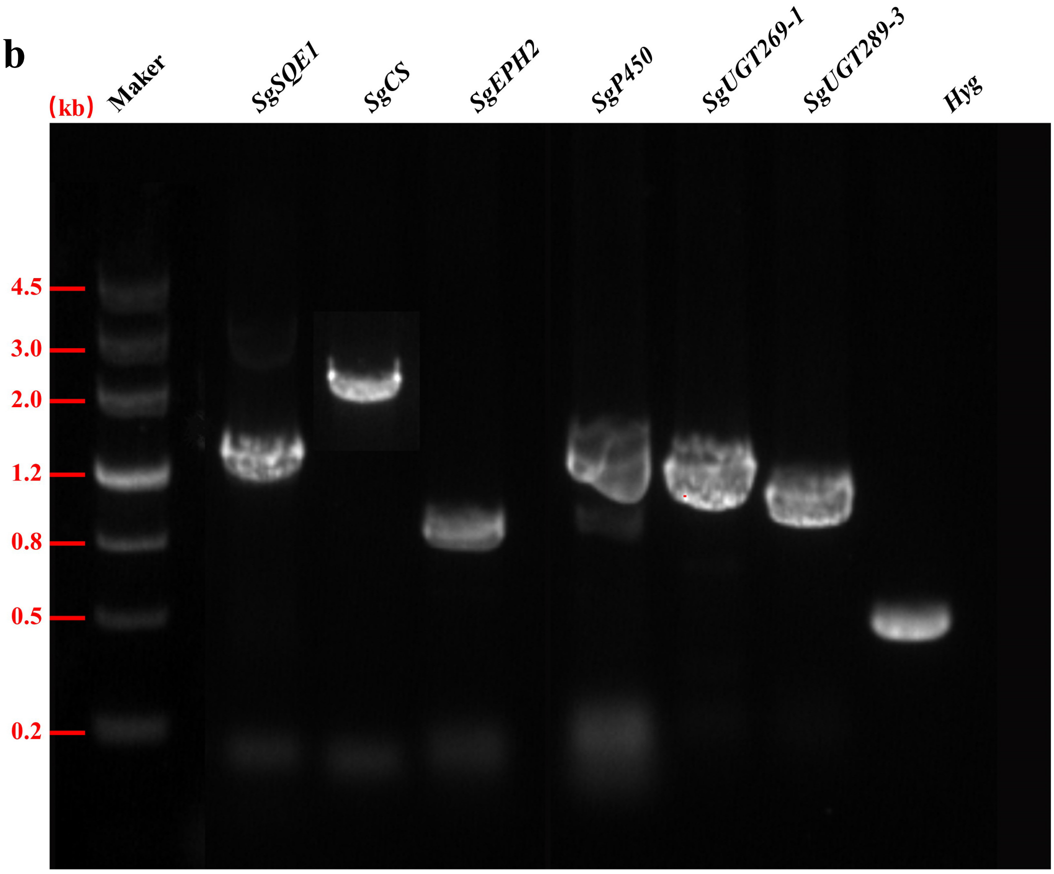
(kb)
Maker
SgSQE1
SgCS
SgEPH2
SgP450
SgUGT269-1
SgUGT289-3
Hyg
4.5
3.0
2.0
1.2
0.8
0.5
0.2

Supplementary Fig S3. Transient accumulation of MIII and mogroside IIE (MII-E) in the leaves of cucumber. a) Transient total ion chromatograms of mogrosides III and MII-E in the leaves of cucumber. **b)** Transient EICs of mogrosides III and MII-E in the leaves of cucumber. The black arrows indicate the peak of MIII and MIIE.

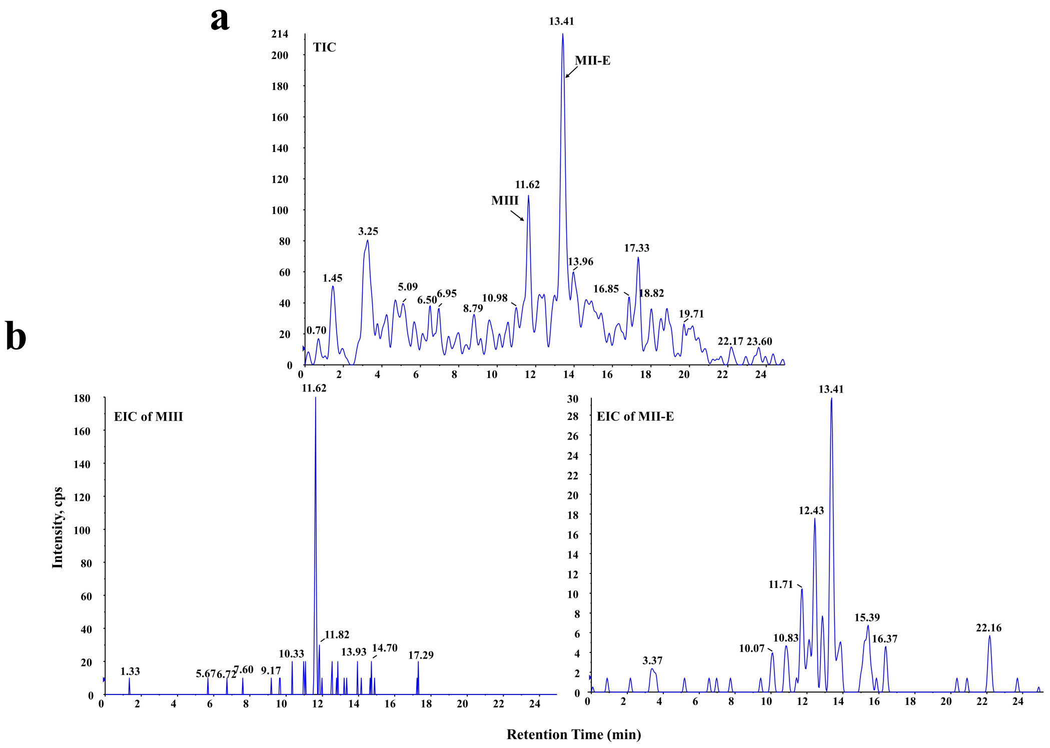

Supplementary Fig S4. Mogrol production in transgenic cucumber and tomato lines. a) dentification of mogrol in the transgenic cucumber line U1. **b)** Accumulation of mogrol in transgenic cucumber line U1. **c)** Identification of mogrol in the transgenic tomato lines S8 and S17. **d)** Accumulation of mogrol in transgenic tomato lines S8 and S17. Black arrow indicates the peak of mogrol. n.d., not detected. The data are presented as the mean values ± SDs, n = 3 biologically independent samples.

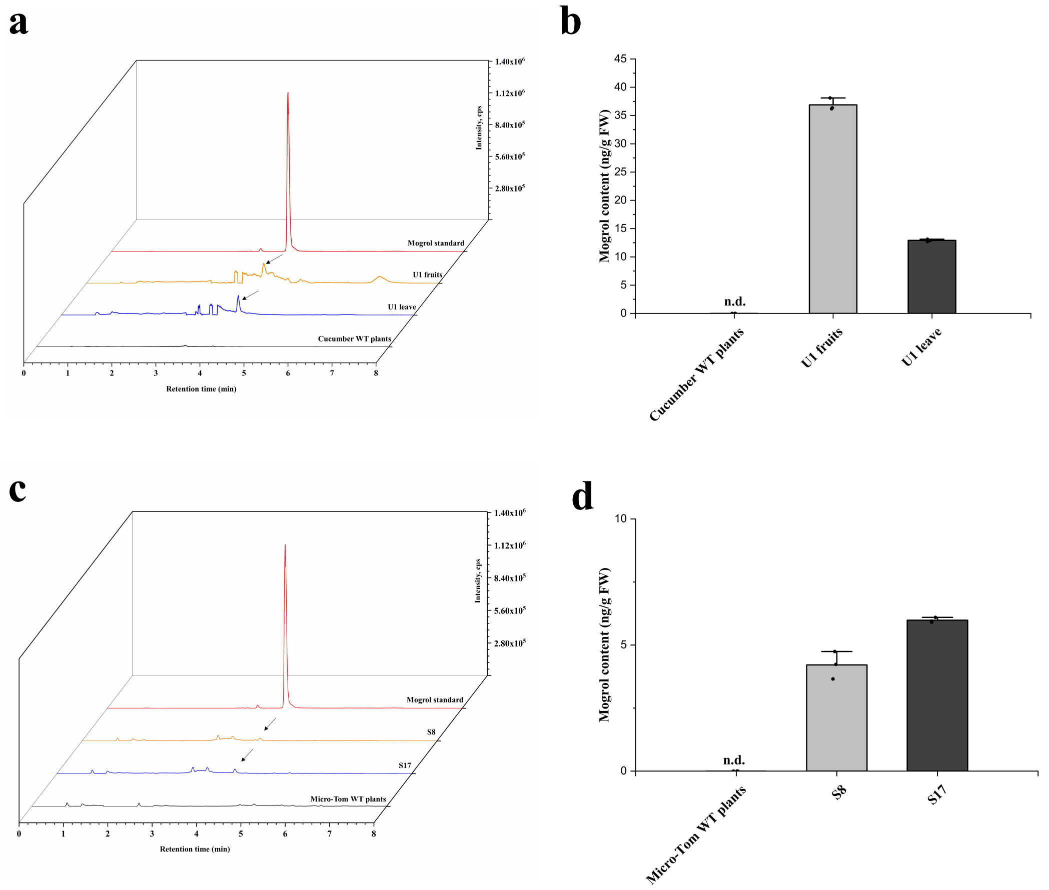

Supplementary Fig S5. The MS and MS/MS spectra of mogrosides for standards and transgenic cucumber plant U1. a) Mogroside V. **b)** Siamenoside I. **c)** Mogroside III. **d)** Mogroside II-E. **e)** Mogroside I-A1.

a

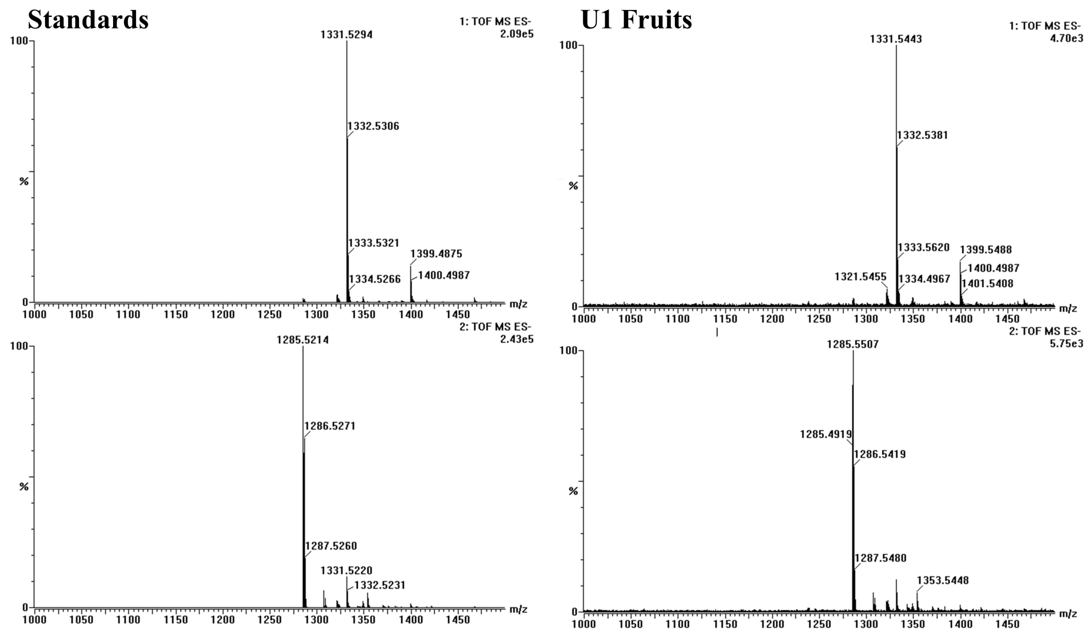

b

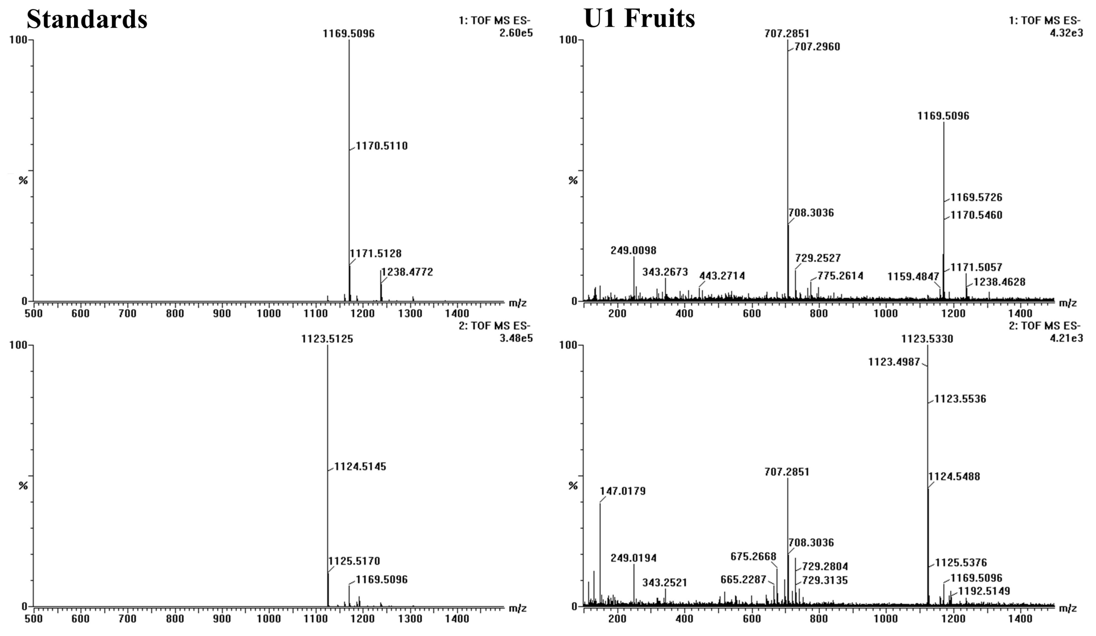

c

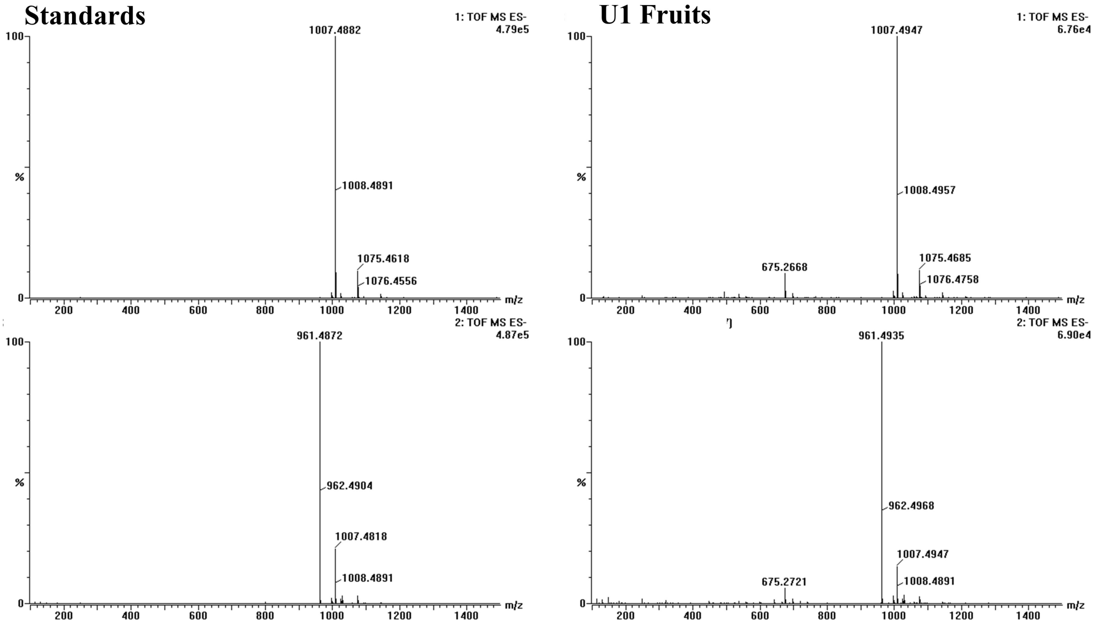
Standards
U1 Fruits
1: TOF MS ES-
4.79e5
1007.4882
1008.4891
1075.4618
1076.4556
1: TOF MS ES-
6.76e4
1007.4947
1008.4957
675.2668
1075.4685
1076.4758
2: TOF MS ES-
4.87e5
961.4872
962.4904
1007.4818
1008.4891
2: TOF MS ES-
6.90e4
961.4935
962.4968
1007.4947
1008.4891
675.2721
%
m/z

d

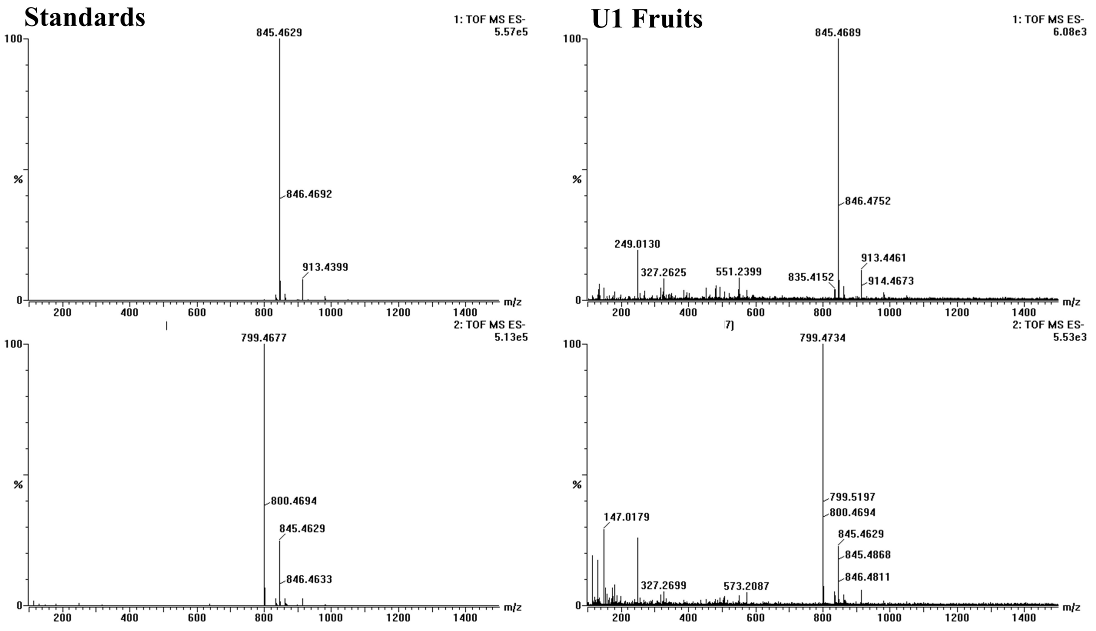
Standards
U1 Fruits
1: TOF MS ES-
5.57e5
845.4629
846.4692
913.4399
1: TOF MS ES-
6.08e3
845.4689
846.4752
249.0130
327.2625
551.2399
835.4152
913.4461
914.4673
2: TOF MS ES-
5.13e5
799.4677
800.4694
845.4629
846.4633
2: TOF MS ES-
5.53e3
799.4734
799.5197
800.4694
845.4629
845.4868
846.4811
147.0179
327.2699
573.2087
%
m/z

e

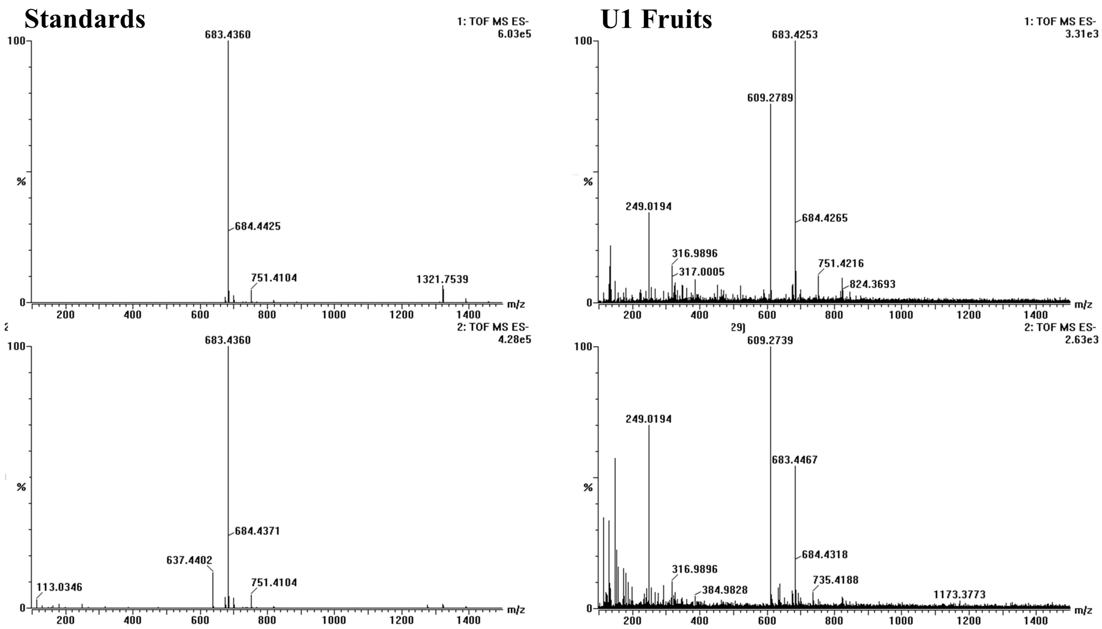
Standards
U1 Fruits
1: TOF MS ES-
6.03e5
683.4360
684.4425
751.4104
1321.7539
3.31e3
683.4253
609.2789
249.0194
684.4265
316.9896
317.0005
751.4216
824.3693
2: TOF MS ES-
4.28e5
683.4360
684.4371
637.4402
113.0346
751.4104
2.63e3
609.2739
249.0194
683.4467
684.4318
316.9896
384.9828
735.4188
1173.3773
%
m/z

Supplementary Figure S6. The UPLC-ESI-QTOF-MS/MS analysis of mogrosides for standards and transgenic Micro-Tom transgenic plant S10. a) The total ion chromatogram of mogrosides in Micro-Tom transgenic plant S10 and standards. MIA-1, MII-E, MIII, SI, and MV represented the mogroside I-A1, mogroside II-E, mogroside III, siamenoside I, and mogroside V, respectively. **b)** The MS and MS/MS spectra of mogroside V in Micro-Tom transgenic plant S10 and standards. **c)** The MS and MS/MS spectra of Siamenoside I. **d)** The MS and MS/MS spectra of Mogroside III. **e)** The MS and MS/MS spectra of Mogroside II-E. **f)** The MS and MS/MS spectra of Mogroside I-A1.

a

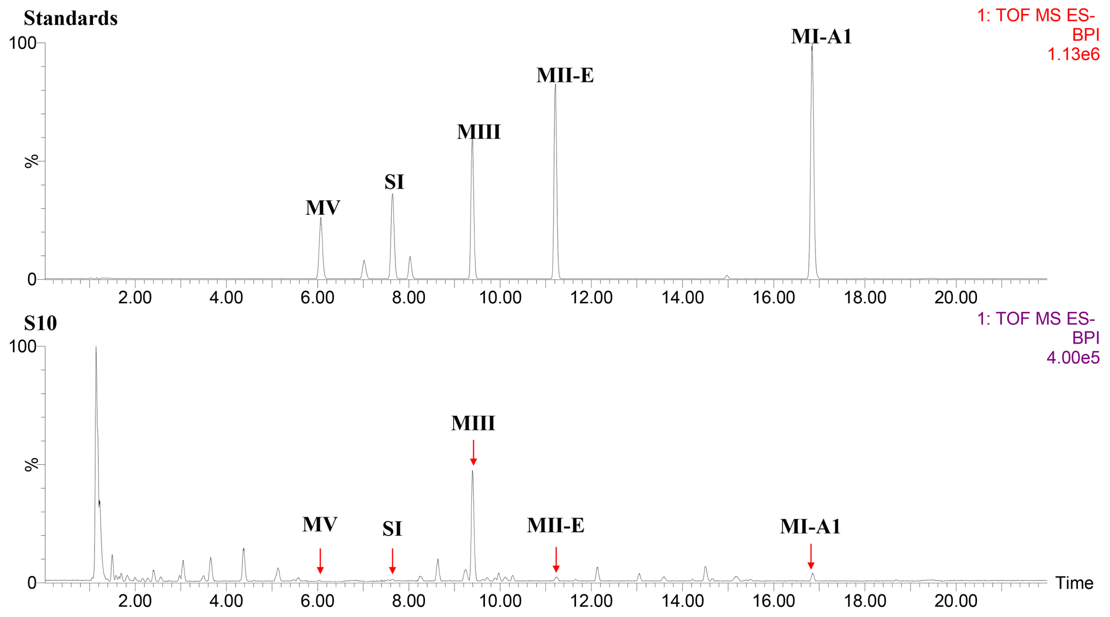

b

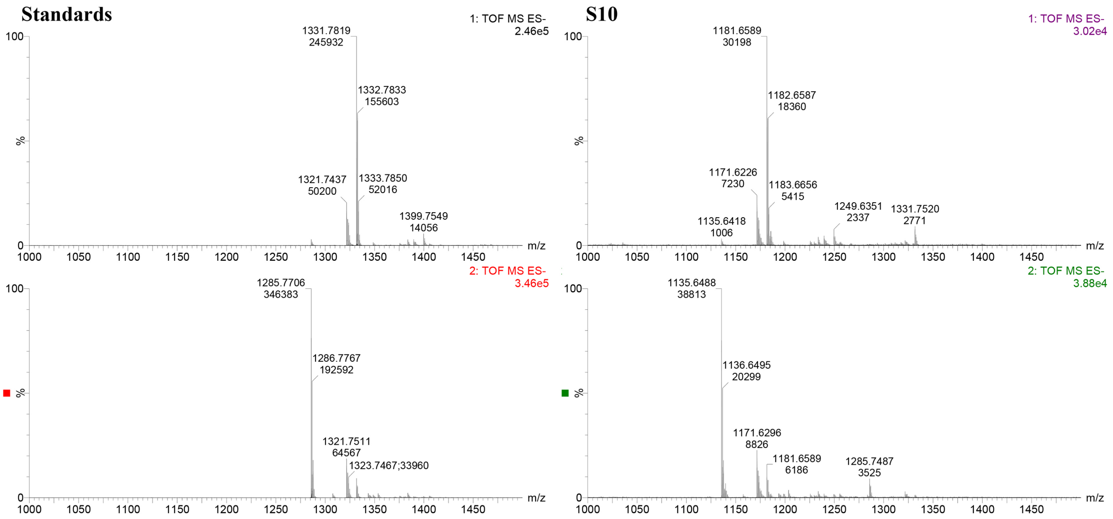

Standards
1: TOF MS ES-
2.46e5
1331.7819
245932
1332.7833
155603
1321.7437
50200
1333.7850
52016
1399.7549
14056
2: TOF MS ES-
3.46e5
1285.7706
346383
1286.7767
192592
1321.7511
64567
1323.7467;33960
S10
1: TOF MS ES-
3.02e4
1181.6589
30198
1182.6587
18360
1171.6226
7230
1183.6656
5415
1135.6418
1006
1249.6351
2337
1331.7520
2771
2: TOF MS ES-
3.88e4
1135.6488
38813
1136.6495
20299
1171.6296
8826
1181.6589
6186
1285.7487
3525
%
m/z

c

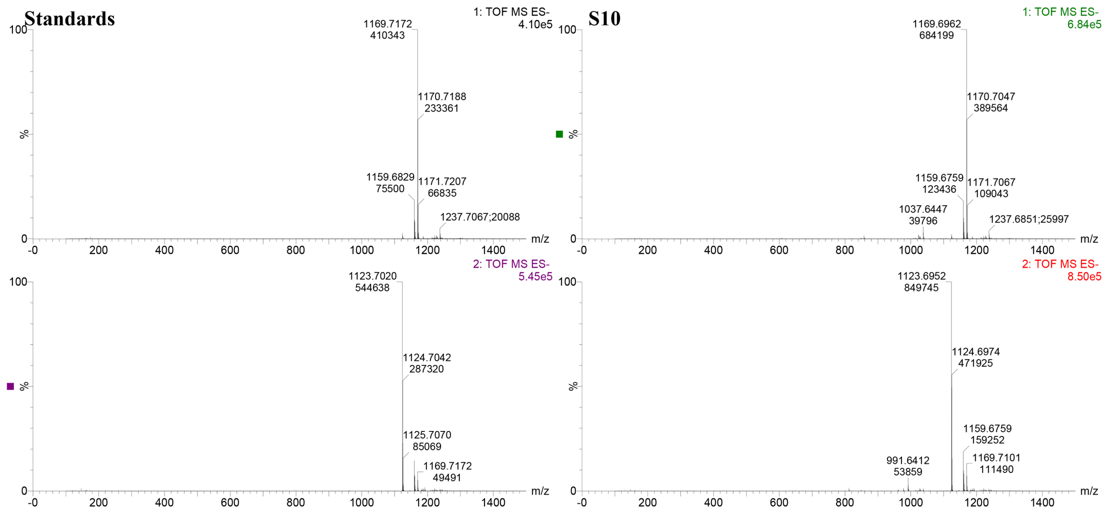

Standards
1: TOF MS ES-
4.10e5
1169.7172
410343
1170.7188
233361
1159.6829
75500
1171.7207
66835
1237.7067;20088
2: TOF MS ES-
5.45e5
1123.7020
544638
1124.7042
287320
1125.7070
85069
1169.7172
49491
S10
1: TOF MS ES-
6.84e5
1169.6962
684199
1170.7047
389564
1159.6759
123436
1171.7067
109043
1037.6447
39796
1237.6851;25997
2: TOF MS ES-
8.50e5
1123.6952
849745
1124.6974
471925
1159.6759
159252
1169.7101
111490
991.6412
53859
%
m/z

d

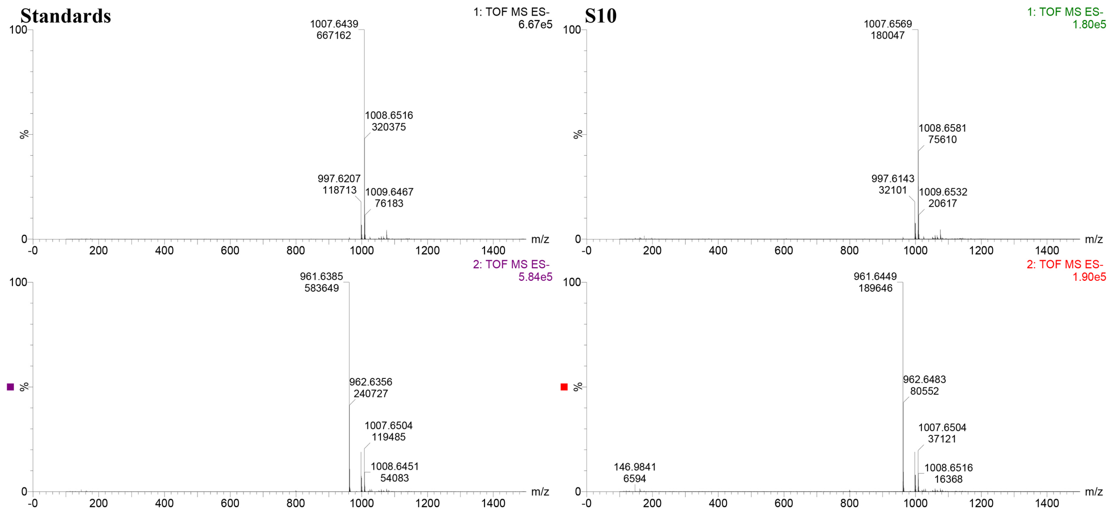

Standards
1: TOF MS ES-
6.67e5
1007.6439
667162
1008.6516
320375
997.6207
118713
1009.6467
76183
2: TOF MS ES-
5.84e5
961.6385
583649
962.6356
240727
1007.6504
119485
1008.6451
54083
S10
1: TOF MS ES-
1.80e5
1007.6569
180047
1008.6581
75610
997.6143
32101
1009.6532
20617
2: TOF MS ES-
1.90e5
961.6449
189646
962.6483
80552
1007.6504
37121
146.9841
6594
1008.6516
16368
m/z
%

e

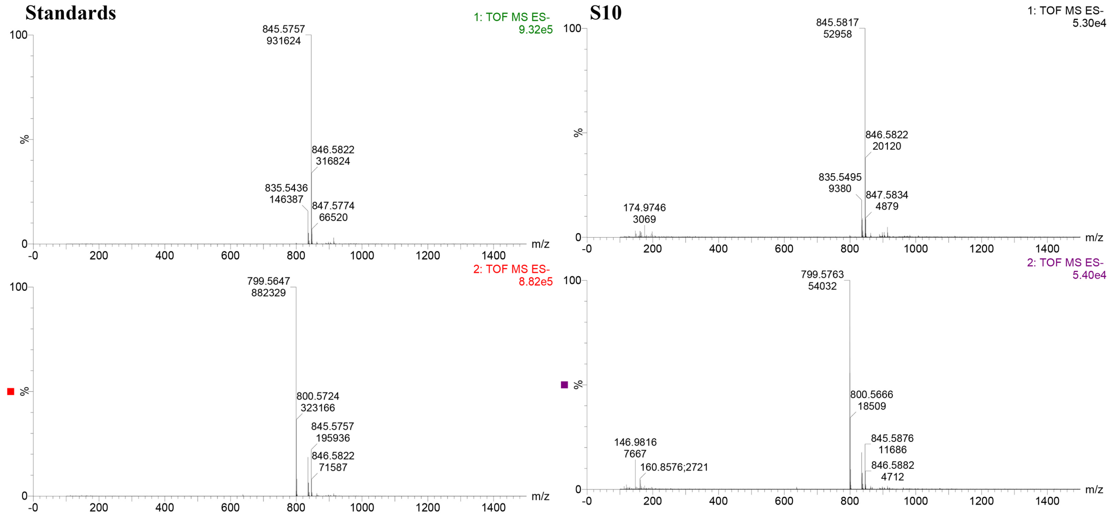

Standards
1: TOF MS ES-
9.32e5
845.5757
931624
846.5822
316824
835.5436
146387
847.5774
66520
2: TOF MS ES-
8.82e5
799.5647
882329
800.5724
323166
845.5757
195936
846.5822
71587
S10
1: TOF MS ES-
5.30e4
845.5817
52958
846.5822
20120
835.5495
9380
847.5834
4879
174.9746
3069
2: TOF MS ES-
5.40e4
799.5763
54032
800.5666
18509
845.5876
11686
146.9816
7667
160.8576;2721
846.5882
4712
m/z
%

f

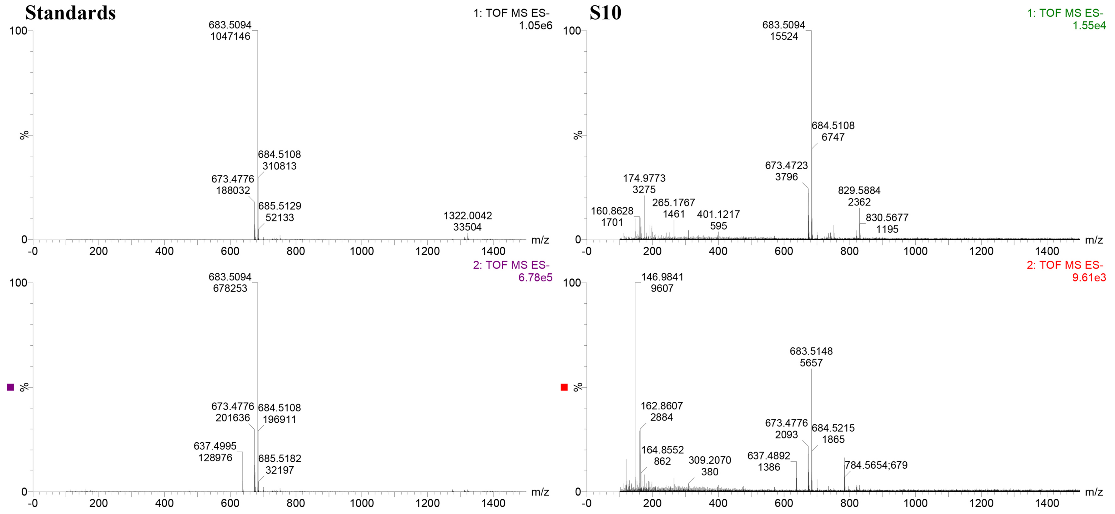

Standards
1: TOF MS ES-
1.05e6
683.5094
1047146
684.5108
310813
673.4776
188032
685.5129
52133
1322.0042
33504
2: TOF MS ES-
6.78e5
683.5094
678253
673.4776
201636
684.5108
196911
637.4995
128976
685.5182
32197
S10
1: TOF MS ES-
1.55e4
683.5094
15524
684.5108
6747
673.4723
3796
174.9773
3275
829.5884
2362
160.8628
1701
265.1767
1461
401.1217
595
830.5677
1195
2: TOF MS ES-
9.61e3
146.9841
9607
683.5148
5657
162.8607
2884
673.4776
2093
684.5215
1865
164.8552
862
309.2070
380
637.4892
1386
784.5654;679
%
m/z

Supplementary Figure S7. unprocessed blots.

related to Figure 2c, 5b and Supplementary Figure S2b.

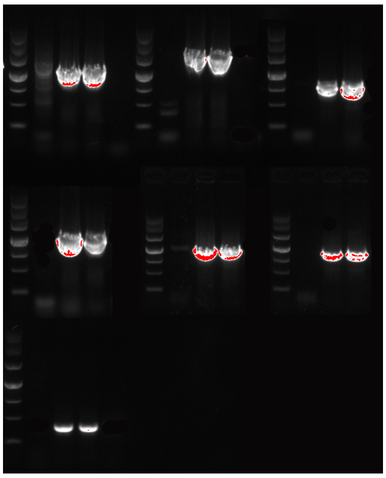

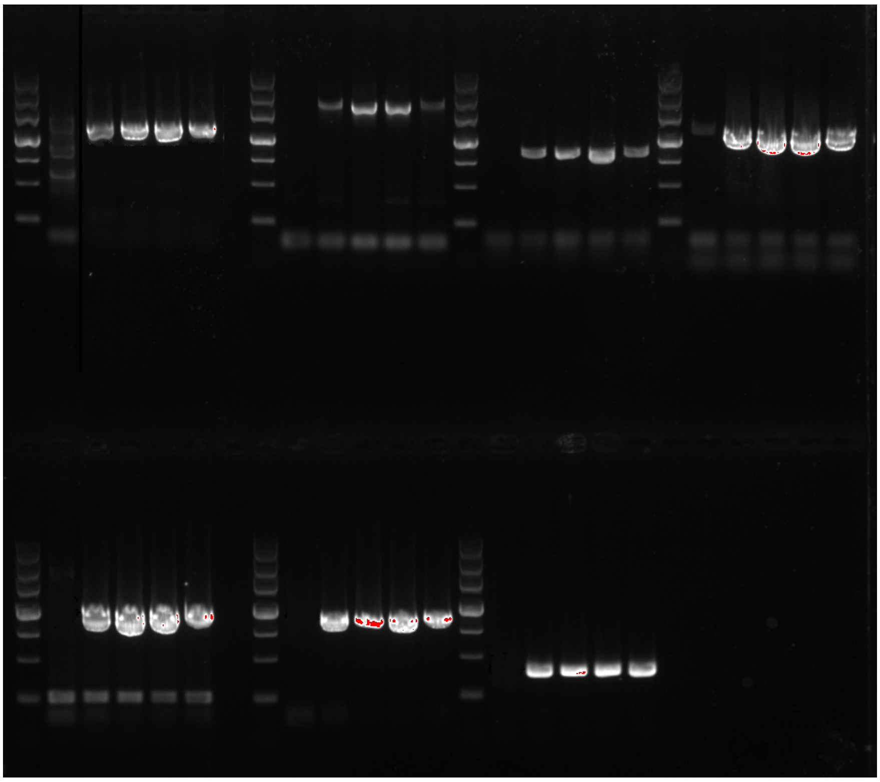

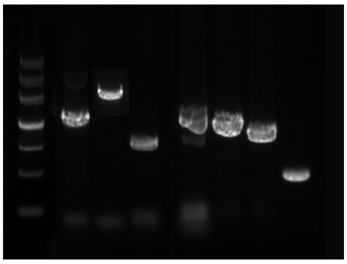

Supplementary Table S1. The data of the cucumber transformation experiment.

ID	Hyg resistance	PCR detection (Number of transgenes)	qRT-PCR	HPLC-MS/MS analysis
**U1**	R	6 (*SgSQE-1, SgCS*, *SgEPH2*, *SgP450*, *Sg269-1*, *Sg289-3*)	OE	MI-A1, MII-E, MIII, SI, MV
**U2**	R	3 *(SgCS*, *SgEPH2*, *Sg289-3*)	ND	ND
**U3**	R	4 (*SgEPH2*, *SgP450*, *Sg269-1*, *Sg289-3*)	ND	ND
**U4**	R	2 (*SgEPH2*, *Sg289-3*)	ND	ND
**U5**	R	4 *(SgCS*, *SgEPH2*, *SgP450*, *Sg289-3*)	ND	ND

[a] R denotes Hygromycin resistance; E denotes Over-expression; ND denotes Not detection.

Supplementary Table S2. Primers used for promoter activity assay

Primer	Sequences (5' to 3')
**121-*AtUBQ10*-F**	gaccatgattacgccaagcttGTCGA**CGAGTCAG**TAATAAACG
**121-*AtUBQ10*-R**	ggactgaccacccggggatccCTGTTAATCAGAAAAACTCAG
**121-*AtPD7*-F**	gaccatgattacgccaagcttATAGGCAACCGTGGACTTCTTC
**121-*AtPD7*-R**	ggactgaccacccggggatccTTGAGGCTAGGTTTTAGTAGTGAAGAA

[a] The homology arm sequences are noted in red; All restriction enzyme sites are underlined.

Supplementary Table S3. Primers used for multigene vector construction

Primers for single gene expression cassette construction

Primer	Sequences (5' to 3')
**PBI121-SgCS-F**	acgggggactctagaggatccATGTGGAGGTTAAAGGTCGGAGC
**PBI121-SgCS-R**	cgatcggggaaattcgagctcTCAAACACTGGTGGACTTCTATAATAAG
**PBI121-SgEPH2-F**	acgggggactctagaggatccATGGAAAACATCGAACACAC
**PBI121-SgEPH2-R**	cgatcggggaaattcgagctcTAACAGAACTTAGAGAAGAAATC
**PBI121-SgP450-F**	acgggggactctagaggatccATGTGGACTGTCGTGCTC
**PBI121-SgP450-R**	cgatcggggaaattcgagctcTCATTCCTTGGGTGTGAACTTCACATGT
**PBI121-SgUGT289-3-F**	acgggggactctagaggatccATGGATGCTGCCCAACAA
**PBI121-SgUGT289-3-R**	cgatcggggaaattcgagctcTCATATTTTAAGCAAGAGAGAAAT
**PBI121-PD7-F**	gaccatgattacgccaagcttATAGGCAACCGTGGACTTCTTCA
**PBI121-PD7-R**	tgatccaccatTTGAGGCTAGGTTTTAGTAGTGAAG
**PD7-SgSQE1-F**	ctagcctcaaATGGTGGATCAGTGCGCGTT
**PD7-SgSQE1-R**	tcttcatcttcatatTTAAACGATTGGCTTAAACAC
**SgSQE1-Thsp-F**	aatcgtttaATATGAAGATGAAGATGAAATATTTG
**SgSQE1-Thsp-R**	aaaacgacggccagtgaattcCTTATCTTTAATCATATTCCATA
**PBI121-UBQ10-F**	gaccatgattacgccaagcttGTCGACGAGTCAGTAATAAACGGC
**PBI121-UBQ10-R**	cgaggttgcaccatCTGTTAATCAGAAAAACTCAGATTAATCG
**UBQ10-SgUGT289-3-F**	taacagATGGTGCAACCTCGGGTACTG
**UBQ10-SgUGT289-3-R**	ccaacatgggagtccaagTTAAAATTTATATGGTTTCAATTTTTTGA
**SgUGT289-3-Tmas-F**	ttttaaCTTGGACTCCCATGTTGGCA
**SgUGT289-3-Tmas-R**	aaaacgacggccagtgaattcGATAATTTATTTGAAAATTCATAAGAAAAGC

[a] The homology arm sequences are noted in red; All restriction enzyme sites are underlined.

Primers for double-gene expression cassette construction

Primer	Sequences (5' to 3')
SQE1-2-F	gaccatgattacgccaagcttATAGGCAACCGTGGACTTCTTCA
SQE1-2-R	gtctcaattgccctttCTTATCTTTAATCATATTCCATA
CS-2-F	ttactagatcAAAGGGCAATTGAGACTTTTCAA
CS-2-R	aaaacgacggccagtgaattcCAGTGAATTCCCGATCTAGTAACATAG
269-2-F	gaccatgattacgccaagcttGTCGACGAGTCAGTAATAAACGGC
269-2-R	ttcttcttgtcaGATAATTTATTTGAAAATTCATAAGAAAAGC
289-2-F	aataaattatcTGACAAGAAGAAAATCTTCGTCAAC
289-2-R	aaacgacggccagtgaattcTTAAGTTGGGTAACGCCAGGG

[a] The homology arm sequences are noted in red; All restriction enzyme sites are underlined.

Primers for triple-gene expression cassette construction

Primer	Sequences (5' to 3')
SC-3-F	gaccatgattacgccaagcttATAGGCAACCGTGGACTTCTTCA
SC-3-R	gccctttggtcAAAGCGAAAGGAGCGGGC
EPH-3-F	tttcgctttGACCAAAGGGCAATTGAGACTT
EPH-3-R	aaaacgacggccagtgaattcGTAAAACGACGGCCAGTGAATT
22-3-F	gaccatgattacgccaagcttGTCGACGAGTCAGTAATAAACGGC
22-3-R	tttgatattttATCTAGTAACATAGATGACACCGCG
P450-3-F	gtcatctatgttactagatAAAATATCAAAGATACAGTCTCAGAAGACC
P450-3-R	aaaacgacggccagtgaattcCCCAGTCACGACGTTGTAAAACG

[a] The homology arm sequences are noted in red; All restriction enzyme sites are underlined.

Primers for final gene expression vector construction

Primer	Sequences (5' to 3')
**U22p-4-F**	ctatgacatgattacgaattcGTCGACGAGTCAGTAATAAACGGC
**U22p-4-R**	**tgCTTCAGCAGGCTGAAGTTAGTAGCTCCGCTTCC**CTTATCGTCGTC<br>ATCCTTGTAATCTT
**SCE-4-F**	**aacttcagcctgctgaagCAGGCTGGAGACGTGGAGGAGAACCCTGGACCT**<br>ATGGTGGATCAGTGCGCGTTGG
**SCE-4-R**	acgacggccagtgccaagcttCCGATCTAGTAACATAGATGACACCG

[a] The homology arm sequences are noted in red; All restriction enzyme sites are underlined.

[b] The sequence of P2A polypeptides are noted in green.

Supplementary Table S4. Primers for PCR detection

Primers	Sequences (5' to 3')
***SgSQE2*-F**	ATGGTGGATCAGTGCGCG
***SgSQE2*-R**	TCAAACACTGGTGGACTTCTATAATAAG
***SgCS*-F**	ATGTGGAGGTTAAAGGTCGGAGC
***SgCS*-R**	TTATTCAGTCAAAACCCGATGGC
***SgEPH*-F**	ATGGAAAACATCGAACACAC
***SgEPH*-R**	TAACAGAACTTAGAGAAGAAATC
***SgP450*-F**	ATGTGGACTGTCGTGCTCGG
***SgP450*-R**	TCATTCCTTGGGTGTGAACTTCACATGT
***SgUGT269*-F**	ATGGTGCAACCTCGGGTA
***SgUGT269*-R**	TTAAAATTTATATGGTTTCAATTTT
***SgUGT289*-F**	ATGGATGCTGCCCAACAA
***SgUGT289*-R**	TCATATTTTAAGCAAGAGAGAAATTTCA
**Hyg-F**	TTGGCGACCTCGTATTGGGA
**Hyg-R**	CAAGACCTGCCTGAAACCGAA

Supplementary Table S5. Primers for qRT-PCR analysis

Primers	Sequences (5' to 3')
***SgSQE1*-qF**	GCTTCGACCATCAACACATTG
***SgSQE1*-qR**	TTCCTCCAAGGCTCAAGTAATC
***SgCS*-qF**	GTTGGGTTGAAGATCCCTACTC
***SgCS*-qR**	CCACAACTGGCTCCCATTAT
***SgCYP450*-qF**	GATGTTCGGTGAGGATGCGA
***SgCYP450*-qR**	TGGTGCCGGGAAAATTCAGT
***SgEPH2*-qF**	AGCTTTGGCTCCATGGTTGA
***SgEPH2*-qR**	CCCAGGTTCTGTCAAAGGCT
***SgUGT269-1*-qF**	CCGATTGAAGTAGCGGAAGAA
***SgUGT269-1*-qR**	CCTCAACGAGCTTCGGTATAAA
***SgUGT289-3*-qF**	CAGAGAAGATGTGCGGAAGAA
***SgUGT289-3*-qR**	TCAGCGACCATCTCATCAAAC
***SgUBQ*-qF**	ATAAAAGACCCAGCACCACATTC
***SgUBQ*-qR**	CCCTTGCCGACTACAACATCC
***CsActin2*-qF**	ATTCTTGCATCTCTAAGTACCTTCC
***CsActin2*-qR**	CCAACTAAAGGGAAATAACTCACC
***Leactin*-qF**	CCAGGTATTGCTGATAGAATGAG
***Leactin*-qR**	GAGCCTCCAATCCAGACAC
